# Aging and menopause reprogram osteoclast precursors for aggressive bone resorption

**DOI:** 10.1038/s41413-020-0102-7

**Published:** 2020-07-01

**Authors:** Anaïs Marie Julie Møller, Jean-Marie Delaissé, Jacob Bastholm Olesen, Jonna Skov Madsen, Luisa Matos Canto, Troels Bechmann, Silvia Regina Rogatto, Kent Søe

**Affiliations:** 1 Clinical Cell Biology, Lillebaelt Hospital, University Hospital of Southern Denmark, 7100 Vejle, Denmark; 2 Department of Regional Health Research, University of Southern Denmark, 5230 Odense M, Denmark; 3 Department of Clinical Biochemistry and Immunology, Lillebaelt Hospital, University Hospital of Southern Denmark, 7100 Vejle, Denmark; 4 Clinical Cell Biology, Department of Pathology, Odense University Hospital, 5000 Odense C, Denmark; 5 Department of Clinical Research, University of Southern Denmark, 5230 Odense M, Denmark; 6 Department of Molecular Medicine, University of Southern Denmark, 5230 Odense M, Denmark; 7 Department of Clinical Genetics, Lillebaelt Hospital, University Hospital of Southern Denmark, 7100 Vejle, Denmark; 8 Department of Oncology, Lillebaelt Hospital, University Hospital of Southern Denmark, 7100 Vejle, Denmark; 9 OPEN, Odense Patient data Explorative Network, Odense University Hospital, 5000 Odense C, Denmark

**Keywords:** Bone, Osteoporosis

## Abstract

Women gradually lose bone from the age of ~35 years, but around menopause, the rate of bone loss escalates due to increasing bone resorption and decreasing bone formation levels, rendering these individuals more prone to developing osteoporosis. The increased osteoclast activity has been linked to a reduced estrogen level and other hormonal changes. However, it is unclear whether intrinsic changes in osteoclast precursors around menopause can also explain the increased osteoclast activity. Therefore, we set up a protocol in which CD14^+^ blood monocytes were isolated from 49 female donors (40–66 years old). Cells were differentiated into osteoclasts, and data on differentiation and resorption activity were collected. Using multiple linear regression analyses combining in vitro and in vivo data, we found the following: (1) age and menopausal status correlate with aggressive osteoclastic bone resorption in vitro; (2) the type I procollagen N-terminal propeptide level in vivo inversely correlates with osteoclast resorption activity in vitro; (3) the protein level of mature cathepsin K in osteoclasts in vitro increases with age and menopause; and (4) the promoter of the gene encoding the dendritic cell-specific transmembrane protein is less methylated with age. We conclude that monocytes are “reprogrammed” in vivo, allowing them to “remember” age, the menopausal status, and the bone formation status in vitro, resulting in more aggressive osteoclasts. Our discovery suggests that this may be mediated through DNA methylation. We suggest that this may have clinical implications and could contribute to understanding individual differences in age- and menopause-induced bone loss.

## Introduction

Bone is continuously being turned over and repaired throughout life. This occurs through a process called bone remodeling, consisting of a tight coordination and balance between bone resorption and bone formation.^1,2^ In this process, bone-resorbing osteoclasts (OCs) and bone-forming osteoblasts (OBs) play a central role. To maintain bone mass throughout adulthood, OBs must replace the precise amount of bone removed by OCs. This link between them, necessary to balance out their activities, is termed “coupling”.^3,4^ However, with age, bone resorption slowly begins to exceed new bone formation during remodeling. Women gradually lose bone mass from the age of ~35, but at menopause, the bone resorption rate increases further, the bone formation rate decreases, and consequently, bone loss is accelerated, making women more prone to osteoporosis.^5–7^ The reason for the increase in OC activity has been studied extensively, and a link to a reduced level of estrogen and possibly to an increased level of follicle stimulating hormone has been shown.^8–12^ Bone turnover can be detected using bone biomarkers, such as serum C-terminal telopeptide of type I collagen (CTX) for bone resorption and serum procollagen type I N propeptide (PINP) for bone formation.^13^ In general, the CTX level is significantly elevated in women with osteoporosis (postmenopausal) compared with nonosteoporotic postmenopausal women, while the PINP level is less elevated and may even be reduced.^14^ Thus, these bone biomarkers can reveal the loss of coupling between bone resorption and formation that eventually leads to osteoporosis. Osteoporosis dramatically affects human health as a major cause of fracture worldwide and is strongly associated with both premature death and morbidity, the latter especially in terms of pain and disability.^7^ Osteoporosis is a very common condition and is associated with a substantial healthcare burden.^7^

Previous studies have shown that estrogen affects OCs in both mice and humans. The decrease in estrogen following ovariectomy/menopause triggers the increased expression of macrophage colony-stimulating factor (M-CSF) and receptor activator of nuclear factor kappa-Β ligand (RANKL) in OB-lineage cells.^15–17^ Since M-CSF and RANKL are both key cytokines driving osteoclastogenesis, a drop in estrogen will indirectly boost the formation of OCs. Estrogen also directly affects OCs, e.g., by reducing the expression of cathepsin K (CatK), a key factor in organic bone matrix degradation.^18–20^ These effects are thought to occur due to the presence or absence of estrogen in the microenvironment. However, in recent years, different observations have indicated that factors such as sex, the menopausal status, and age affect the properties of OCs themselves, in a manner unrelated to the microenvironment and presence or absence of ligands/receptors. This has mainly been investigated using OCs differentiated from peripheral blood mononuclear cells (PBMCs)^21–23^ and bone marrow-derived OCs,^23^ which are both widely accepted models for the generation and characterization of human OCs in vitro. Several studies have indicated that in vitro-generated OCs act and/or respond in a sex-dependent manner in humans.^24–28^ In addition, sex-dependent differences in the resorption mode of OCs in vitro have also been suggested.^29^ Aging and/or menopause have also been found to affect OC formation both in vivo and in vitro.^28^ First, the OC progenitor pool has been reported to increase with advancing age in humans^30,31^ and mice.^16,32^ However, in mice, this increase was only observed with stimulation by OB-derived cytokines, such as interleukin-3, granulocyte-macrophage colony-stimulating factor, and M-CSF.^32^ Second, monocytes from ovariectomized (OVX) rats were shown to have the ability to differentiate into mature OCs in vitro independently of M-CSF and RANKL, in contrast to monocytes from non-OVX rats.^33^ Osteoporosis has also been shown to be associated with increased OC formation and bone resorptive properties. When comparing spontaneous OC formation in vitro using PBMCs from postmenopausal osteoporotic patients with PBMCs from age-matched controls, a significant increase in OC formation was found in the osteoporotic patients.^34^ Of note, D’Amelio and coauthors also correlated clinical features (using cells from osteoporotic postmenopausal patients versus cells from healthy age-matched postmenopausal women) with in vitro OC formation.^34^ Thus, there are indications that OC formation and activity in vitro could be more permanently affected by certain in vivo features, such as sex, age, and the menopausal status.

Epigenetic alterations may also be a plausible explanation for linking in vivo features to OC formation/activity in vitro. A general drift in DNA methylation occurs during a lifetime due to aging, environmental influences, and lifestyle, predominantly resulting in DNA hypomethylation.^35,36^ Menopause further accelerates this aging-related DNA hypomethylation^37,38^ and can be reversed by estrogen replacement therapy.^39,40^ Changes in DNA methylation have also been associated with osteoporosis.^39,41–44^ For that reason, we speculate that alterations in DNA methylation may be involved in OC reprogramming by changing the gene expression pattern of key OC genes, making OCs more aggressive. Therefore, we hypothesized that aging and/or menopause will induce long-term changes in OC precursors and that this will increase their ability to create mature OCs and resorb bone.

## Results

### The bone resorption activity of OCs in vitro reflects the in vivo characteristics of donors

**Fig. 1:**
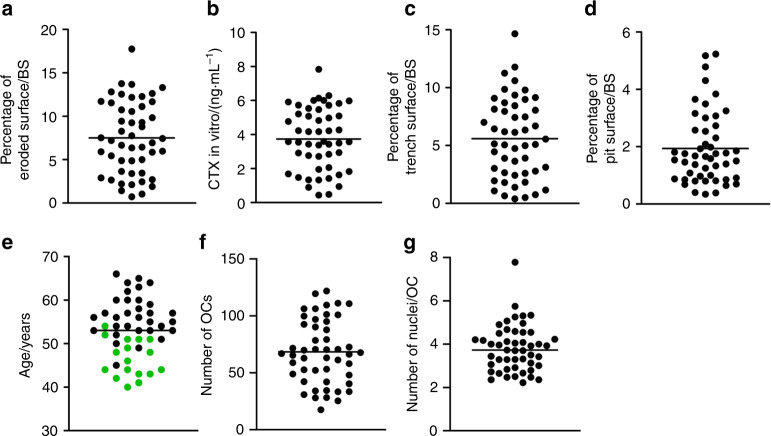
Variation of results obtained using OCs generated from different donors for the given variables: (**a**) percent eroded surface/bone surface (mean = 7.51); (**b**) CTX level in vitro (mean = 3.74); (**c**) percent trench surface/bone surface (mean = 5.60); (**d**) percent pit surface/bone surface (median = 1.66); (**e**) donor age (mean = 53.0); (**f**) mean number of OCs per vision field (mean = 68.4); and (**g**) mean number of nuclei/OC (median = 3.73). In (**e**), green dots indicate premenopausal donors, while black dots indicate postmenopausal donors. Each dot represents the results obtained from OCs generated from an individual donor (*n* = 49)

The demographic information of the 49 included blood donors is shown in Table 1. Figure 1 displays the variation and mean/median of some of the most important variables in this study. Using OC preparations from the blood donors, we found that the level of bone resorption in vitro varied from 0.73% to 17.7% (mean of 7.51%) eroded surface/bone surface (ES/BS) (Fig. 1a). In an attempt to understand what may cause this large variation in OC resorption activity in vitro, we tested the influence of a series of variables. Table 2 shows the optimized multiple linear regression model following a likelihood ratio test for the four dependent variables reflecting the OC bone resorption activity level: percent ES/BS, CTX in vitro (ng·mL^−1^), percent trench surface/BS, and percent pit surface/BS. An initial multiple linear regression model was generated including the following independent variables: CTX level in vivo (ng·mL^−1^), PINP level in vivo (µg·L^−1^), age, menopausal status (pre/post), smoking status (yes/no), medication (yes/no), height (m), weight (kg), number of OCs, number of nuclei/OC, percent trench surface/BS, percent pit surface/BS, and the statistical constant “_cons”. The table shows the variables that best predicted the dependent variable. We found that age, the PINP level in vivo, and the number of nuclei/OC were the best predictors of the total ES, which together explained 59% (*r*^2^ = 0.59) of the observed variation (Table 2). Age (*P* = 0.011), and the number of nuclei/OC (*P* < 0.001) were both positively correlated with the percent ES/BS, while the PINP level in vivo (*P* = 0.045) was inversely correlated (Table 2). Of note, these findings suggest that the in vivo characteristics of donors can influence the bone resorption activity of OCs in vitro, at least with respect to the total ES.

**Table 1:** Demographics of 49 female blood donors

Demographic characteristics	Clinical features	*N*	Median(IQR^a^) [range]	Mean(SD^b^) [range]	%
Age	40–66	49		53.0 (6.7) [40; 66]	100.0
	40–44	8			16.3
	45–49	6			12.2
	50–54	15			30.6
	55–59	11			22.5
	60–66	9			18.4
Menopausal status	Premenopausal	17		46.4 (5.0) [40; 54]	34.7
	Postmenopausal	32		56.5 (4.9) [45; 66]	65.3
Years since menopause		49	3.5 (8.25) [0; 23]		100.0
Smoking status	Nonsmoker	42			85.7
	Smoker	7			14.3
Comorbidities	No	41			83.7
	Yes	8			16.3
	-Hypothyroidism	3			6.1
	-Asthma/Allergy	3			6.1
	-Ulcers	2			4.1
Height/m		49		1.70 (0.6) [1.56; 1.84]	100.0
Weight/kg		49		73.2 (13.35) [55; 124]	100.0
BMI^c^		49		25.4 (4.0) [19.5; 37.8]	100.0

^a^Interquartile range

^b^Standard deviation

^c^Body mass index

**Table 2:** Optimized regression models obtained using multiple linear regression analyses and likelihood ratio tests

Dependent variable	*R*^2^	Independent variable	Coef.	SD	*t*	*P*
Percentage of eroded surface/BS^a^	0.59	Age	1.17	0.06	2.64	0.011
		PINP level in vivo	−0.05	0.02	−2.11	0.041
		Number of nuclei/OC	2.66	0.38	7.06	<0.001
		_cons	−9.13	3.24	−2.81	0.007
CTX level in vitro^b^	0.74	Percentage of eroded surface/BS	0.38	0.04	10.850	<0.001
		CTX level in vivo	1.60	0.83	1.93	0.060
		Menopausal status	0.64	0.33	1.91	0.062
		_cons	−0.04	0.53	−0.08	0.934
Percentage of trench surface/BS^c^	0.61	Age	0.15	0.05	2.95	0.005
		PINP level in vivo	−0.04	0.19	−2.29	0.027
		Number of nuclei/OC	2.21	0.31	7.23	<0.001
		_cons	−8.78	2.63	−3.34	0.002
Percentage of pit surface/BS^d^	0.38	Percentage of trench surface/BS	0.15	0.04	3.53	0.001
		Number of OCs	0.02	0.01	3.73	0.001
		_cons	−0.24	0.45	−0.54	0.590

^a^LR chi^2^ = 2.82, *P* = 0.728, df = 5

^b^LR chi^2^ = 4.90, *P* = 0.557, df = 6

^c^LR chi^2^ = 6.90, *P* = 0.330, df = 6

^d^LR chi^2^ = 3.73, *P* = 0.811, df = 7

In addition, we used measurements of CTX in the conditioned media of bone-resorbing OC cultures as another measure of bone resorption activity. The CTX level in vitro varied from 0.44 to 7.84 ng·mL^−1^, with a mean of 3.68 ng·mL^−1^ (Fig. 1b). However, when using CTX as the dependent variable, none of the in vivo variables showed a significant correlation, although the CTX level in vivo (*P* = 0.060) and the menopausal status (*P* = 0.062) showed nearly significant correlations (Table 2). Finally, a strong positive correlation with the percent ES/BS (*P* < 0.001) was obtained. Thus, variations in the CTX level in vitro do not reflect the in vivo characteristics of donors to the same extent as the total ES/BS.

### OCs making trenches, and not those making pits, are correlated with in vivo characteristics

During analyses of bone resorption, all resorption cavities were subdivided into pits or trenches, as previously defined.^29,45,46^ The percent trench surface/BS varied between 0.39% and 14.66%, with a mean of 5.50%, while pit formation varied from 0.34% to 5.23%, with a median of 1.66% pit surface/BS (Fig. 1c, d). We found that trench formation was best predicted by age (*P* = 0.005), the PINP level in vivo (*P* = 0.027) and the number of nuclei/OC (*P* < 0.001), which together explained 61% (*r*^2^ = 0.61) of the observed variation. In comparison, pit formation was best predicted by the number of OCs (*P* = 0.001) and percent trench surface/BS (*P* = 0.001), explaining only 38% (*r*^2^ = 0.38) of the observed variation. Hence, it is the trench, not the pit, resorption mode that reflects the in vivo characteristics. Individual correlations of total ES/BS, CTX level in vitro, trench surface/BS, and pit surface/BS with donor age are depicted in Supplementary Information 1A–D.

### The activity of OCs in vitro also reflects the donor menopausal status

Both aging and menopause are factors that may affect OC bone resorption activity, but while assessing all assumptions of the linear regression model, high multicollinearity was found among the predictor variables: age and years since menopause. Therefore, to avoid multicollinearity, age was replaced with years since menopause in a separate multiple linear regression model (Table 3), in which the dependent and independent variables included in the initial model were otherwise the same as those shown in Table 2. The observed variation in the percent ES/BS was best predicted by years since menopause (*P* = 0.045) and the number of nuclei/OC (*P* < 0.001) (Table 3). Both parameters were positively correlated with the percent ES/BS, and the optimized model explained 56% (*r*^2^ = 0.56) of the observed variation. This result again shows that the in vivo characteristics of donors can influence the bone resorption activity of OCs in vitro and that both aging and menopause are correlated with more aggressive bone resorption. It made no difference whether age or years since menopause was included in the CTX in vitro model (Table 3). When testing what parameters best predicted the formation of trenches with years since menopause included in the model (Table 3), only the number of nuclei/OC (*P* < 0.001) ended up in the optimized model, explaining 52% (*r*^2^ = 0.52) of the observed variation. In comparison, pit formation was best predicted by the number of OCs and percent trench surface/BS, as observed in Table 2. Thus, it seems that the activity of OCs making trenches, not of those making pits, is responsible for the increase in bone resorption during aging, but neither appears to be responsible for the increase in menopause-related resorption.

**Table 3:** Optimized regression models obtained using multiple linear regression analyses and likelihood ratio tests

Dependent variable	*R*^2^	Independent variable	Coef.	SD	*t*	*P*
Percentage of eroded surface/BS^a^	0.56	Years since menopause	0.15	0.07	2.07	0.045
		Number of nuclei/OC	2.59	0.39	6.67	<0.001
		_cons	−3.12	1.50	−2.07	0.044
CTX level in vitro^b^	0.74	Percentage of eroded surface/BS	0.38	0.04	10.850	<0.001
		CTX level in vivo	1.60	0.83	1.93	0.060
		Menopausal status	0.64	0.33	1.91	0.062
		_cons	−0.04	0.53	−0.08	0.934
Percentage of trench surface/BS^c^	0.52	Number of nuclei/OC	2.31	0.32	7.12	<0.001
		_cons	−3.20	1.28	−2.50	0.016
Percentage of pit surface/BS^d^	0.38	Percentage of trench surface/BS	0.15	0.04	3.53	0.001
		Number of OCs	0.02	0.01	3.73	0.001
		_cons	−0.24	0.45	−0.54	0.590

^a^LR chi^2^ = 6.56, *P* = 0.364, df = 6

^b^LR chi^2^ = 4.90, *P* = 0.557, df = 6

^c^LR chi^2^ = 13.16, *P* = 0.106, df = 8

^d^LR chi^2^ = 6.09, *P* = 0.529, df = 7

### The promoter of *TM7SF4* is less methylated in older women than in younger women

Since the level of resorption correlated with the age and menopausal status of the donor, even after 9 days of in vitro differentiation from monocyte to mature multinucleated OC, we investigated whether monocytes are “reprogrammed” as women age. A plausible mechanism for this “reprogramming” may involve alterations in the DNA methylation level of key OC genes. Based on the work of de la Rica et al.^47^ we selected the genes encoding CatK (*CTSK*) and DC-STAMP (*TM7SF4*) for examination. DC-STAMP is considered a key regulator of osteoclastogenesis.^48,49^ CatK is the most important proteinase required for collagen degradation by OCs during bone resorption.^50–52^ Genomic DNA was isolated from mature OCs, just prior to their reseeding on bone slices, to perform bisulfite pyrosequencing to evaluate the CpG methylation pattern of their promoters.

**Fig. 2:**
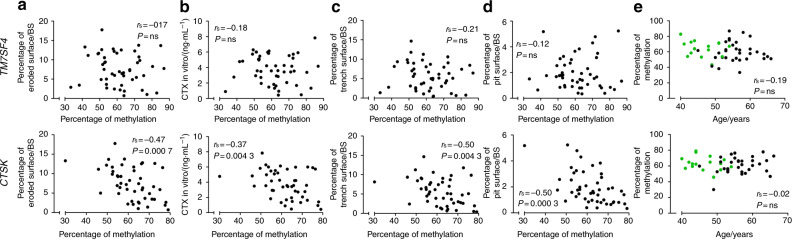
Average DNA methylation of CpGs in the promoter of the *TM7SF4* gene, encoding DC-STAMP, (top row) and that of the *CTSK* gene, encoding cathepsin K, (bottom row) compared with (**a**) percent eroded surface/bone surface, (**b**) CTX level in vitro, (**c**) percent trench surface/bone surface, (**d**) percent pit surface/bone surface, and (**e**) donor age (years). In (**e**), green dots indicate premenopausal donors, while black dots indicate postmenopausal donors. Statistical correlation analyses were performed using Spearman’s rank correlation (*r*_s_). Each dot represents the results obtained from OCs generated from an individual donor (*n* = 49)

**Fig. 3:**
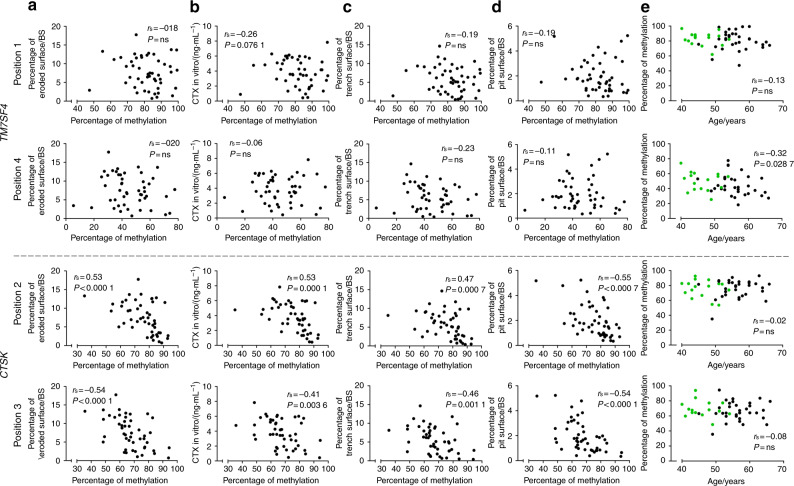
DNA methylation status of selected CpG sites in the promoter of the *TM7SF4* gene, encoding DC-STAMP, (top rows) and that of the *CTSK* gene, encoding cathepsin K, (bottom rows) compared with (**a**) percent eroded surface/bone surface, **(b**) CTX level in vitro, (**c**) percent trench surface/bone surface, (**d**) percent pit surface/bone surface, and (**e**) donor age (years). In (**e**), green dots indicate premenopausal donors, while black dots indicate postmenopausal donors. Statistical correlation analyses were performed using Spearman’s rank correlation (*r*_s_). Each dot represents the results obtained from OCs generated from an individual donor (*n* = 49). The selected CpG sites are indicated as “positions”. Only relevant positions were selected to be shown in this figure. The correlations of positions that are shown here are significant or near significant. Positions that are not shown here did not correlate with any of the abovementioned variables

**Fig. 4:**
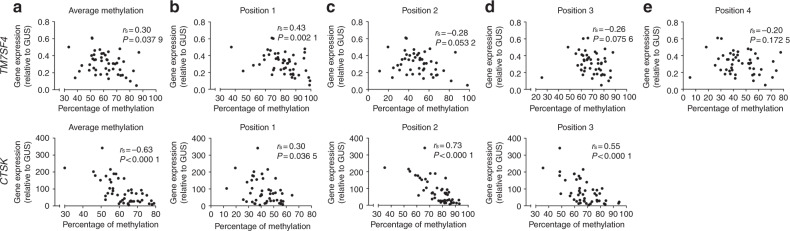
Comparison of the expression of the *TM7SF4* gene, encoding DC-STAMP, (top row) and the *CTSK* gene, encoding cathepsin K, (bottom row) with the DNA methylation status of their promoter regions based on (**a**) the average or (**b**–**e**) the individual CpG sites. Statistical correlation analyses were performed using Spearman’s rank correlation (*r*_s_). Each dot represents the results obtained from OCs generated from an individual donor (*n* = 49). The CpG sites are indicated as “positions”

**Fig. 5:**
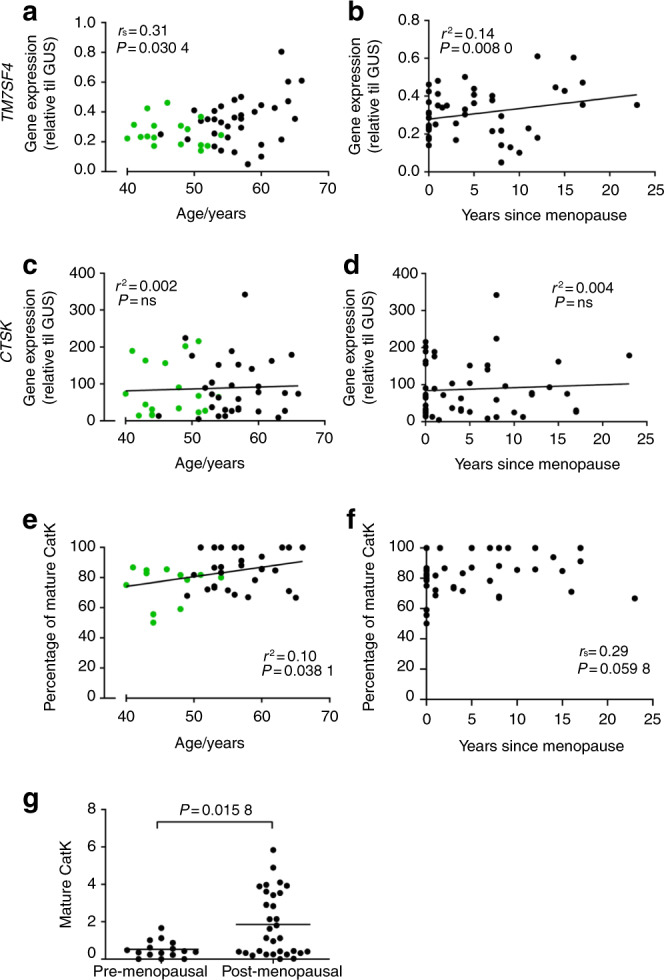
Comparison of the gene expression level of the *TM7SF4* gene, encoding DC-STAMP, with (**a**) the donor age, or (**b**) the number of years since menopause (*n* = 48). Comparison of the gene expression level of the *CTSK* gene, encoding cathepsin K, with (**c**) the donor age, or (**d**) the number of years since menopause (*n* = 49). Comparison of the percentage of mature cathepsin K (of the total cathepsin K for each donor) within each OC culture, as determined by Western blotting with (**e**) the donor age (*n* = 46) and (**f**) the number of years since menopause (*n* = 46). (**g**) Comparison of the relative amount of mature cathepsin K (normalized to the β-actin level) between pre- and postmenopausal women as determined by Western blotting (*n* = 46). In **a**, **c**, and **e**, green dots indicate premenopausal donors, while black dots indicate postmenopausal donors. Statistical correlation analyses were performed using either Spearman’s rank correlation (*r*_s_) or Pearson’s correlation (*r*^2^). Comparisons between two groups were performed using the Mann–Whitney test. One data point in a was excluded using the ROUT outlier test (*Q* = 2%). Each dot represents the results obtained from OCs generated from an individual donor

The average methylation status of the four CpGs evaluated for *TM7SF4* did not correlate with any of the variables related to in vitro bone resorption (Fig. 2a–d). In addition, no significant correlation was found with the number of nuclei/OC (data not shown) or the donor age (Fig. 2e). Comparing the methylation status of the four individual CpGs of *TM7SF4* with the variables related to in vitro bone resorption (Fig. 3a–d) showed no correlations, though the methylation of position 1 showed a nearly significant correlation with the CTX level in vitro (*P* = 0.076 1). However, when comparing the methylation status of the individual CpGs with the donor age, a significant inverse correlation for the CpG at position 4 was found (*P* = 0.028 7) (Fig. 3e). The DNA methylation levels were compared with the gene expression levels to verify that variations in DNA methylation (as shown in Figs. 2 and 3) also had an impact on gene expression. We found an inverse correlation between the gene expression and the average methylation status of CpGs in the *TM7SF4* promoter (*P* = 0.037 9) (Fig. 4a). When investigating individual CpG sites, position 1 was significantly correlated with gene expression (*P* = 0.002 1), positions 2 and 3 were nearly significant (*P* = 0.053 2, *P* = 0.075 6), and position 4 showed no correlation (Fig. 4b–e). In addition, we also found that the gene expression of *TM7SF4* increased with both age (*P* = 0.030 4) and the number of years since menopause (*P* = 0.008 0) (Fig. 5a, b). Moreover, the gene expression level of *TM7SF4* was positively correlated with the number of nuclei/OC (*P* = 0.018 0) (data not shown), which in turn was the strongest predictor of bone resorption activity in vitro (Tables 2 and 3). Altogether, our data suggest that methylation within the promoter region of the *TM7SF4* gene decreases with age, increasing gene expression, and the fusion potential of OCs in vitro.

### The methylation status of the *CTSK* gene is associated with the level of bone resorption in vitro, while the protein level of mature CatK correlates with the donor age

The average methylation status of *CTSK* showed an inverse correlation (*P* = 0.000 7) with the percent ES/BS (Fig. 2a), CTX level in vitro (*P* = 0.008 8) (Fig. 2b), percent trench surface/BS (*P* = 0.004 3) (Fig. 2c), and percent pit surface/BS (*P* = 0.000 3) (Fig. 2d). No correlation was found with the donor age (Fig. 2e). Similar results were found for individual CpG sites in the promoter region of *CTSK* (Fig. 3). The methylation status of the CpGs at positions 2 and 3 of the *CTSK* promoter correlated with the percent ES/BS (*P* < 0.000 1 and *P* < 0.000 1), CTX level in vitro (*P* < 0.000 1 and *P* = 0.003 6), percent trench surface/BS (*P* = 0.000 7 and *P* = 0.001 1), and percent pit surface/BS (*P* < 0.000 1 and *P* < 0.000 1) (Fig. 3a–d), while the methylation status of no individual sites correlated with the donor age (Fig. 3e). In summary, the methylation status of the *CTSK* gene is significantly correlated with all parameters related to in vitro bone resorption. We found no correlation between *CTSK* gene expression and donor age or the number of years since menopause (Fig. 5a, b). Notably, the level of mature CatK expression in the OCs (as determined by Western blotting) was significantly higher in postmenopausal women than in premenopausal women (Fig. 5e), and the percentage of mature CatK increased with age (Fig. 5c) but not with the number of years since menopause (Fig. 5d). The average DNA methylation level of the *CTSK* promoter was found to be inversely correlated with the gene expression level (*P* < 0.000 1) (Fig. 4a). This was also the case for all individual CpGs in the *CTSK* promoter (Fig. 4b–d). In addition, the average methylation status of *CTSK* was inversely correlated with the protein level of mature CatK (Supplementary Information 4A), and accordingly, the gene expression level of *CTSK* was positively correlated with the level of mature CatK (Supplementary Information 4B).

## Discussion

In this study, more than a 20-fold variation in the resorption activity of OCs among donors was observed in vitro, and interestingly, this variation was correlated with several in vitro and in vivo characteristics. We found that aging and/or the number of years since menopause were correlated with the properties of OCs derived from PBMCs. This means that the in vivo characteristics of donors can predict the bone resorption activity of OCs in vitro. We also found that a single CpG mapped in the promoter region of the *TM7SF4* gene was less methylated in older women than in younger women. *TM7SF4*/DC-STAMP is considered a master regulator of osteoclastogenesis.^48^

A possible explanation for the observed correlation between the resorptive activity of in vitro-generated OCs and the age/menopausal status of the respective donors could be that monocytes are epigenetically “reprogrammed” as women age and/or enter menopause. This would allow OCs generated in vitro from these monocytes to “remember” the age/menopausal status of the donor and resorb bone more actively. Such “reprogramming” appears plausible, as a general drift in DNA methylation has been found to occur during life due to aging, environmental influences, and lifestyle, predominantly resulting in hypomethylation.^35,36^ In addition, menopause has been found to further accelerate this aging-related hypomethylation,^37,38^ which can be reversed by estrogen replacement therapy.^39,40^ Such alterations have also been linked to osteoporosis.^39,41–44^ Our current study indicates that the mechanism behind the observed “reprogramming” may actually involve alterations in the DNA methylation level of key OC genes, such as *TM7SF4* and *CTSK*. This supports the idea that a reduced estrogen level may trigger an alteration in DNA methylation that “reprograms” OCs through their precursors (monocytes) to be more aggressive.

Apart from changes in the DNA methylation level, there may also be other, but not necessarily contradictory, explanations for our results. For example, a change in the pool of CD14^+^ pre-OCs (monocytes) has been observed during aging and/or menopause. Monocytes originate from a myeloid precursor in the bone marrow and circulate in the bloodstream. They are precursors for tissue macrophages, OCs, and dendritic cells.^53^ Different monocyte subpopulations were found to differ with respect to their function both in mice^54^ and humans.^55^ These monocyte subpopulations were found to be altered as a consequence of aging.^56^ With age, an increase in “nonclassical” CD14^+^DC16^+^ monocytes was observed at the expense of classical CD14^+^DC16^−^ monocytes, resulting in monocytes with different expression levels of several surface proteins and chemokine receptors and possibly altering the activity of subsequent OCs. An indication of this was found in multiple myeloma patients since the fraction of CD14^+^CD16^+^ monocytes was significantly increased in patients with osteolytic bone disease compared with the controls.^57^ An alternative explanation for the correlation between the resorptive activity of in vitro-generated OCs and the age/menopausal status of the respective donors could be an age-related change in the expression of circulating miRNAs, known to function as RNA silencers and posttranscriptional regulators of gene expression.^58,59^

In support of the hypothesis that changes in DNA methylation reprogram key OC genes, we found increased *TM7SF4* expression levels according to age and the number of years since menopause, while the *CTSK* gene did not show a correlation. It is also relevant to note that the gene expression level of *TM7SF4* correlated positively with the number of nuclei/OC, which in turn was the strongest predictor of bone resorption activity in vitro. In addition, the number of nuclei/OC correlated with *CTSK* expression. Although neither the DNA methylation status nor the gene expression of *CTSK* was correlated to the age/menopausal status of the donors, we found that the protein level of mature CatK in the OCs was significantly higher in postmenopausal women than in premenopausal women and that the percentage of mature CatK (in percent of total CatK for each individual donor) increased significantly with age. The observed correlation between the mature CatK level and aging appears to be induced through a different mechanism than DNA methylation. The fact that it is specifically the percentage of mature CatK (within each osteoclast culture) that increases with age suggests that it is induced through a mechanism that regulates the activation of CatK, rather than the expression level of CatK. Nevertheless, this needs to be further investigated. Altogether, these data support that age and menopause may reprogram OC precursors for more aggressive bone resorption through alterations in DNA methylation and through an mechanism of CatK activation. Thus, we may have found a link between our in vivo and in vitro parameters.

Some studies have suggested correlations between clinical features and OCs in vitro. D’Amelio et al. compared the occurrence of spontaneous (no addition of M-CSF and RANKL) OC formation in vitro using PBMCs from osteoporotic patients with PBMCs from age-matched controls and found a significant increase in OC formation but not in resorptive activity in osteoporotic patients.^34^ In addition, Jevon et al. demonstrated increased osteoclastic resorption activity on dentine slices using PBMCs from osteoporotic patients compared to controls, without any increase in OC formation (using M-CSF, and RANKL).^60^ Compared with our study, in that study, the authors investigated the effect of osteoporosis on osteoclastic bone resorption activity and not the effect of age/menopause. Jevon and colleagues also linked OC activity with clinical features when comparing OC formation and activity between age-matched men and women.^28^ When coculturing OCs with OB-like cells, they found that postmenopausal females and males of comparable age showed similar levels of both osteoclastogenesis and dentine degradation. In comparison with age-matched males, premenopausal women showed similar levels of osteoclastogenesis but less dentine degradation, indicating sex-dependent differences in OC activity when estrogen is present.^28^ However, in contrast to our findings, they did not find an age- or menopause-induced difference among women.^28^ In our study, experiments were only performed using samples from females. Further analyses are therefore needed to clarify whether a similar age effect is observed in men.

Variation in the shape of excavations has been observed in several studies both in vitro and in vivo.^61–63^ Recently, it was demonstrated through time-lapse analyses^64^ that they reflect two different modes of OC resorption: the intermittent pit mode and the continuous trench mode.^29,46^ Furthermore, sex affected the relative balance between these resorption modes, since OCs derived from females primarily resorbed in pit mode, while OCs derived from males resorbed in trench mode to a higher extent.^29^ In our present study, we found that OCs making trenches, not those making pits, responded to the in vivo characteristics of the female donors. The PINP level correlated inversely with the resorptive activity of OCs in vitro, especially when OCs were in the aggressive trench mode. At first glance, this inverse correlation seems counterintuitive. However, it is in line with a series of observations indicating an inverse relationship between the levels of mature CatK/aggressiveness of resorption and osteoprogenitor recruitment/bone formation.^65–70^ Overall, these observations highlight that the distinction between the trench and pit resorption modes is relevant to keep in mind when analyzing osteoclastic bone resorption activity both in vitro and in vivo. When assessing the level of bone resorption by measuring the CTX level in vitro, we found only a nearly significant correlation with the in vivo parameters (CTX level in vivo and menopausal status). This may appear puzzling, but it is worth noting that in vitro pits can skew the CTX measurements because collagen is not released from pits to the same extent as from trenches.^45,46,71,72^

The strongest predictor for resorptive activity in vitro was the mean number of nuclei/OC in vitro. The mean numbers of OCs and nuclei/OC were quantified before an identical number of cells were seeded per bone slice for all donors. This means that the variables, i.e., the number of OCs and the number of nuclei/OC, are indicators of the precursors’ ability to differentiate into mature multinucleated OCs. The nucleation status of OCs has previously been described to correlate with bone resorption in vitro.^73^ In addition, we found that the nucleation status was correlated with trench-mode bone resorption. The nucleation status of the OCs was not relevant to pit formation. Overall, this could indicate that OCs with more nuclei are more inclined toward making trenches, while those with fewer nuclei are inclined toward making pits.

We obtained promising results by analyzing the selected CpG methylation sites for *TM7SF4* and *CTSK* based on the work of de la Rica et al.^47^ However, it may be considered a limitation that the DNA methylation analyses were performed using mature multinucleated OCs. To obtain a more direct link between in vivo and in vitro characteristics, it could be even more relevant to investigate the methylation status of monocytes. Finally, a comprehensive analysis of the global methylation profile of monocytes is required to better understand the effects of epigenetics on OC activity in vitro. DNA methylation analyses have the potential to identify individuals at high risk of developing osteoporotic fractures. In the near future, comprehensive DNA methylation profiles could be used as an additional risk assessment tool and thereby further promote individualized treatment strategies for osteoporotic patients.

In conclusion, our data demonstrate the following: (1) age and menopausal status correlate with more aggressive bone resorption in vitro; (2) OCs making trenches, not those making pits, reflect in vivo characteristics; (3) the in vitro protein level of mature CatK in OCs increases with menopause and donor age; and (4) the *TM7SF4* promoter is less methylated in older women than in younger women. This suggests that monocytes are “reprogrammed” as women age and/or enter menopause and become increasingly more active, resorbing bone in trench mode. The DNA methylation level of key OC genes, such as *TM7SF4*, could contribute to the reprogramming process. An improved understanding of the mechanisms leading to bone loss is important to better target the treatment of osteoporosis and reduce the risk of fractures. We suggest that our findings may have implications for understanding individual differences in age/menopause-induced osteoporosis, which might be utilized to personalize the treatment of osteoporosis.

## Materials and methods

### Study population and samples

Fifty healthy female blood donors between 40 and 66 years of age were recruited from the blood donor corps, Vejle Hospital, Vejle, Denmark. The exclusion criteria were prior bisphosphonate treatment and fractures within the last two years. Following a regular blood donation (500 mL), samples were fractioned, and the buffy coat was collected for further use. In addition, fasting blood samples were collected by a standard venipuncture procedure ~2 weeks (mean: 12.8, median: 14 days) after study inclusion (blood donation). To obtain serum, the blood was allowed to clot at room temperature before the samples were centrifuged at 2 000 × *g* for 10 min. Immediately after centrifugation, the serum phase was stored at −80 °C until use, and the samples had not been thawed prior to analysis. Using questionnaires, each participant provided information on lifestyle and medical history, including age, menopausal status (0 = postmenopausal, 1 = premenopausal), years since menopause (years), height (m), weight (kg), smoking habits (0 = nonsmoker, 1 = smoker), comorbidities (0 = no, 1 = yes) and medications (0 = no, 1 = yes). As the participants were recruited from the existing pool of blood donors, they were all considered healthy due to the very strict health requirements for donating blood. Eight of the donors had minor medical conditions; three donors had hypothyroidism, two donors had ulcers and three donors had asthma and/or allergies. The basic characteristics of the donor population are described in Table 1. One donor was excluded due to technical problems during the purification of CD14^+^ monocytes.

### In vitro generation of human OCs

CD14^+^ monocytes were purified from the buffy coat of each donor sample by centrifugation through Ficoll-Paque (Amersham, GE Healthcare, Little Chalfont, UK) and then isolated by immunomagnetic separation 48 h after sampling, as previously described.^74^ Briefly, PBMCs were suspended in PBS containing 0.5% BSA and 2 mmol·L^−1^ EDTA, and CD14^+^ cells were purified using antihuman CD14 magnetic particles (BD Biosciences, San Jose, CA, USA) according to the supplier’s instructions. Cells were seeded at a density of 5 × 10^6^ cells/T75 culture flasks (Greiner, Frickenhauser, Germany) in αMEM (Invitrogen, Carlsbad, CA, USA) containing 10% FBS (Sigma-Aldrich, St. Louis, MO, USA) and 25 ng·mL^−1^ M-CSF (R&D System, Abingdon, UK). Cells were differentiated into mature OCs over nine days with M-CSF and RANKL (R&D System), as previously described.^45,75^ After nine days of maturation, 12 systematic and evenly distributed images of OCs from each donor were taken using a CKX41 microscope with an SC30 camera (Olympus Corporation, Shinjuku, Tokyo, Japan). These images were used to manually count the number of OCs (with ≥2 nuclei) and the mean number of nuclei/OC. A representative example from three different donors is displayed in Supplementary Information 3A.

### Bone resorption assays

Mature OCs were detached from culture flasks using Accutase (Biowest BW, Nuaillé, France) and then reseeded on 0.4-mm-thick bovine cortical bone slices (BoneSlices.com, Jelling, Denmark) at a density of 50 000 cells/bone slice (5 bone slices/donor) in 96-well plates. OCs were cultured for 72 h with M-CSF and RANKL, and then conditioned media was stored at −80 °C for later analysis (CTX measurement). Bone resorption was visualized by toluidine blue staining, and the percent ES/BS was analyzed by light microscopy using a 100-point grid (Pyser-SGI, Edenbridge, UK), as previously described.^45^ Representative examples of resorption patterns for three different donors are displayed in Supplementary Information 3B. A comparison of the mean number of nuclei/OC with the percent ES/BS for each donor is also indicated (Supplementary Information 3C). All resorption surfaces were subdivided into pits and trenches.^76^ Pits were defined as a single excavation, circular in appearance, with well-defined edges and a ratio between the length and width of the excavation not exceeding two. A trench was defined as an elongated and continuous excavation, with well-defined edges and ratio of length to width of at least two, in accordance with published definitions.^29,64^ During analyses, the observer was fully blinded with respect to any information about the blood donor.

### CTX-I and PINP measurements

Routine diagnostic analyses were carried out at the Department of Biochemistry and Immunology, Vejle Hospital, accredited by the Danish Accreditation Fund according to the ISO 15189 standard. Measurement of fasting serum CTX and PINP levels and CTX levels in conditioned media were all performed by routine chemiluminescence immunoassays, according to the manufacturer’s instructions (Cobas e602 analyzer, Roche Diagnostics, Denmark). All samples were stored at −80 °C until analysis, and none of the serum samples had been thawed prior to analysis.

### DNA methylation analyses using pyrosequencing

The methylation status of CpGs mapped in the promoters of *CTSK* (3 CpGs) and *TM7SF4* (4 CpGs) was evaluated using pyrosequencing. Genomic DNA was extracted using the QIAamp DNA Mini Kit (Qiagen, Valencia, CA, USA) following the instructions of the supplier. The resulting DNA concentration and purity were determined using a Nanodrop 1000 Spectrophotometer (Thermo Scientific, Waltham, MA, USA). The DNA (500 ng) was bisulfite-converted using the EZ DNA Methylation-Gold™ Kit (Zymo Research, Irvine, CA, USA). Briefly, after bisulfite conversion, 10 ng of DNA was used for amplification using the PyroMark PCR Kit (Qiagen); the PCR products were inspected using D1000 ScreenTape on an Agilent TapeStation (Agilent Technologies, Glostrup, Denmark) and then sequenced on a PyroMark Q24 system (Qiagen). The sequences of the primers used for PCR and pyrosequencing of each CpG region are described in Supplementary Information 2.

### Droplet digital RT-PCR

Cells from each donor were lysed, and RNA was extracted using the TRIzol Plus RNA Purification Kit (Invitrogen), as previously described.^77^ cDNA was generated from 500 ng of the extracted RNA and the iScript cDNA Synthesis Kit (Bio-Rad, Hercules, CA, USA). The copy number concentrations were measured by droplet digital RT-PCR using a QX100™ Droplet Digital™ PCR system (Bio-Rad). The absolute quantification of PCR targets was analyzed using QuantaSoft™ software version 1.3.2.0 (Bio-Rad). The expression of the target genes was normalized to that of the reference gene *GUS*. All TaqMan primer sets were used according to the supplier’s instructions (Applied Biosystems) as follows: GUS: Hs99999908_m1 (VIC-MGB), CATK: Hs00166156_m1 (FAM-MGB), and DC-STAMP: Hs00166156_m1 (FAM/MGB).

### Western blot analyses

Western blotting was performed as previously described (using 6 µg of protein extract)^75^ using polyclonal rabbit-αhCatK antibody (Abcam, Cambridge, UK, ab49893) as the primary antibody and HRP-coupled anti-rabbit antibody (ECL WB system, RPN 2108, GE Healthcare) as the secondary antibody. The chemiluminescence signals were detected using a ChemiDoc MP Imaging System (Bio-Rad). Then, the membranes were stripped, incubated with monoclonal mouse-αhβActin antibody (Sigma-Aldrich, A2228, clone AC-74) and detected as described above. Quantification of Western blot data was performed using Image Lab software (Bio-Rad, version 6.1.0.). For comparison of the total protein level of mature CatK among donors, the CatK levels were normalized to the β-actin level. In addition, the percentage of mature CatK (in percent of total CatK for each individual donor) was assessed.

### Statistics

Multiple linear regression analyses, relevant model assumptions, and likelihood ratio tests were performed using STATA/SE, version 15 (StataCorp, College Station, TX, USA). To ensure that no multicollinearity existed among predictor variables, variance inflation factor diagnostics was performed. To find the best predicting variables, the least significant variables were removed from the dataset step by step. Likelihood ratio tests were used to ensure that no predictive value was lost from the dataset in the process. This was done until no further removal of variables was permitted according to the likelihood ratio tests, and the final model was then accepted as the optimal model. All graphs were created and related statistical analyses were performed using GraphPad Prism software, version 5 (GraphPad software, San Diego, CA, USA). Correlations were evaluated using Spearman’s rank correlation (r_s_) or Pearson’s correlation (noted in figures with the coefficient of determination (*r*^2^)), and the result was considered significant if the *P* value was <0.05. Comparisons between two groups were performed using the Mann–Whitney test. One data point in Fig. 5a was excluded using the ROUT outlier test (*Q* = 2%). Each dot represents the results obtained from OCs generated from an individual donor. All figures were created using CorelDRAW X5 (Corel Corporation, Canada).

### Study approval

Human blood donations and blood samples were all collected at the Department of Clinical Biochemistry and Immunology (blood donor corps) at Lillebaelt Hospital under a protocol approved by The Scientific Ethical Committee for the Region of Southern Denmark with approval number 20150059. Written informed consent was received from each participant prior to inclusion in the study. During inclusion, all participants were given a distinct ID number and were subsequently only identified by this number.

## Supplementary information

Clean version

Marked-up

## Data Availability

The datasets generated and/or analyzed during the current study are available from the corresponding author on reasonable request.
