# Supplementary material for: Aging and menopause reprogram osteoclast precursors for aggressive bone resorption
Source: Bone Res. 2020 Jul 1;8:27. doi: 10.1038/s41413-020-0102-7 (PMC7329827; doi:10.1038/s41413-020-0102-7)
Supplement: Supplementary file 1 — Clean version [file 41413_2020_102_MOESM1_ESM.docx]

**Aging and menopause reprogram osteoclast precursors for aggressive bone resorption**

**Running title: Age reprograms pre-OCs for aggressive bone erosion**

*Anaïs Marie Julie Møller, Jean-Marie Delaissé, Jacob Bastholm Olesen^,^ Jonna Skov Madsen, Luisa Matos Canto, Troels Bechmann, Silvia Regina Rogatto, Kent Søe*

**Supplementary Information**


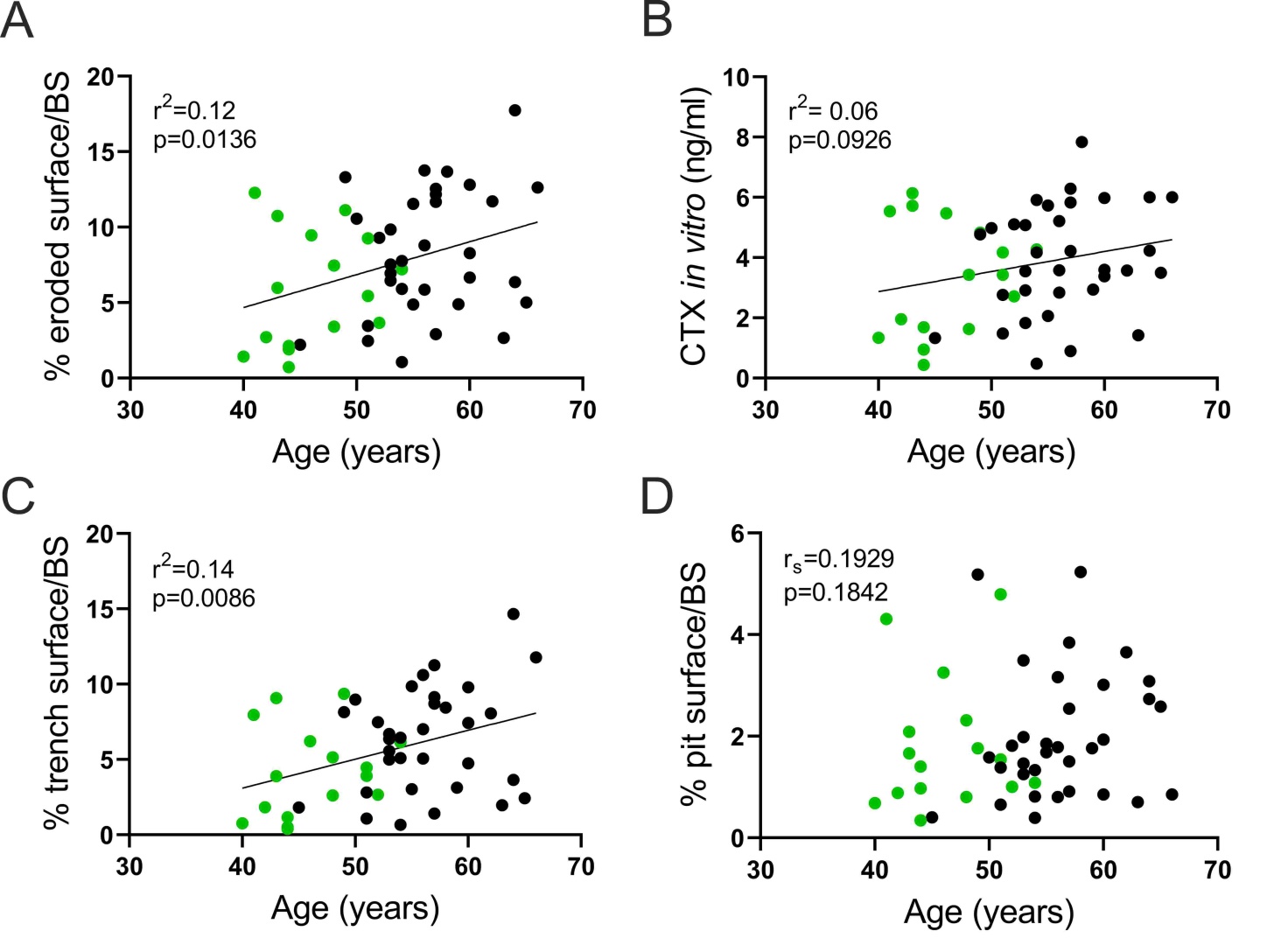


Supplementary Information 1 Correlation of donor age with A) % eroded surface/BS, B) CTX levels in conditioned media (ng/ml), C) % trench surface/BS, and D) % pit surface/BS. Statistical correlation analyses were performed using either Spearman's rank correlation (r_s_) or Pearson's correlation (r^2^). Green dots represent pre-menopausal donors, while black dots represent post-menopausal donors. Each dot reflects the results obtained from OCs generated from an individual donor (n=49).


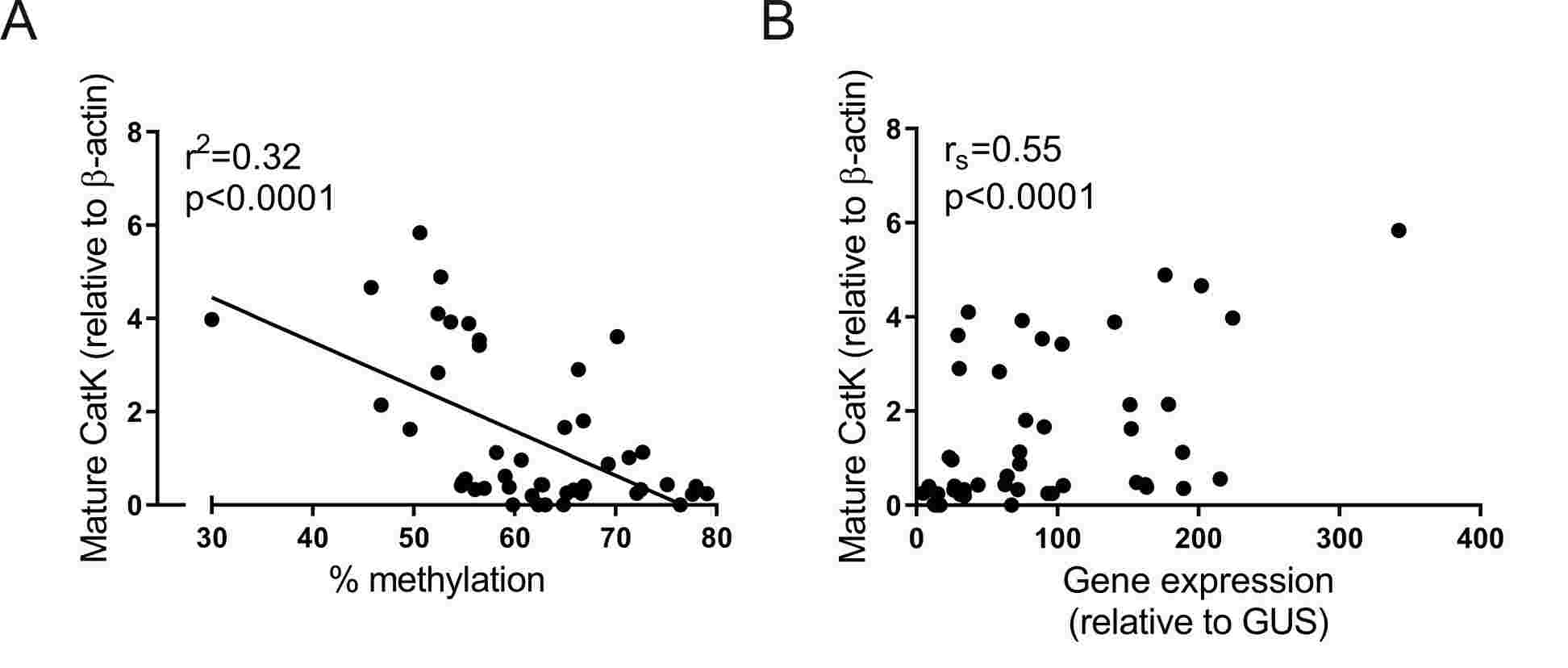


**Supplementary Information 2** A) Correlation of the average methylation status of the *CTSK* gene with the quantity of mature cathepsin K among donors. B) Correlation of the gene expression of the *CTSK* gene with the quantity of mature cathepsin K among donors. Statistical correlation analysis was performed using either Spearman's rank correlation (r_s_) or Pearson's correlation (r^2^). Each dot represents the results obtained from OCs generated from an individual donor (n=46).


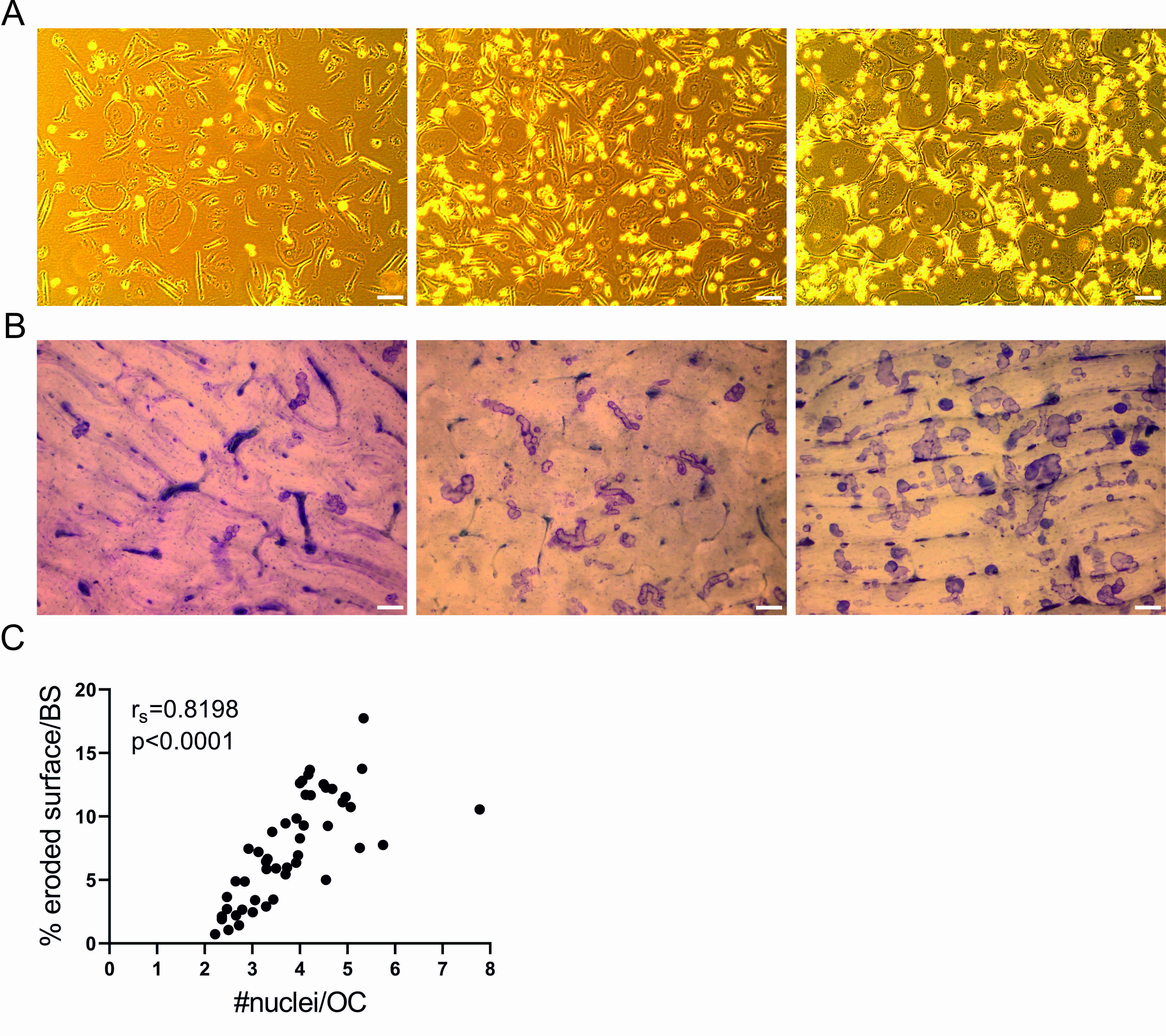


Supplementary Information 3 Representative images of A) cell cultures after 9 days of differentiation with RANKL (scale bar = 100µm), B) cortical bovine bone slices after 3 days of resorption (50,000 OCs/bone slice) from 3 different donors (scale bar = 100µm), C) comparison of the mean number of nuclei/OC with the percent eroded surface/BS for each donor. Statistical correlation analysis was performed using Spearman's rank correlation (r_s_). Each dot represents the results obtained from OCs generated from an individual donor (n=49).

**Supplementary Information 4** Primers description used for pyrosequencing^[[1]](#footnote-1)^

| **Primer** | **Sequence 5´>3'** |
| --- | --- |
| CTSK_Forward | GTGGGTATATTATTAGGGGTG |
| CTSK_Reverse | [Btn^[[2]](#footnote-2)^]CTCCTCCTCTTACCCAAATT |
| CTSK_Sequencing | GTTAGATTTATTTTAT |
|  |  |
| TM7SF4_Forward | TGTTTGGGGTTATGAGTGTAG |
| TM7SF4_Rreverse | [Btn]TTACCCTCACTCCCATACT |
| TM7SF4_Sequencing | GGTTATGAGTGTAGAGG |

1. The annealing temperature for all PCR primers was 60°C [↑](#footnote-ref-1)
2. Biotinylated [↑](#footnote-ref-2)
